# Suspicion of respiratory tract infection with multidrug-resistant Enterobacteriaceae: epidemiology and risk factors from a Paediatric Intensive Care Unit

**DOI:** 10.1186/s12879-017-2251-x

**Published:** 2017-02-21

**Authors:** Hanna Renk, Lenja Stoll, Felix Neunhoeffer, Florian Hölzl, Matthias Kumpf, Michael Hofbeck, Dominik Hartl

**Affiliations:** 1 Dept. of Paediatric Cardiology, Pulmology and Intensive Care Medicine, University Children’s Hospital Tübingen, Hoppe-Seyler Str. 1, Tübingen, 72076 Germany; 2 Institute of Medical Microbiology and Hygiene, University of Tübingen, Elfriede-Aulhorn-Str.6, Tübingen, 72076 Germany; 3 Dept. of Paediatrics, Pediatric Infectious Diseases, Immunology & Pneumology/Cystic fibrosis, University Children’s Hospital Tübingen, Hoppe-Seyler Str. 1, Tübingen, 72076 Germany

## Abstract

**Background:**

Multidrug-resistant (MDR) infections are a serious concern for children admitted to the Paediatric Intensive Care Unit (PICU). Tracheal colonization with MDR Enterobacteriaceae predisposes to respiratory infection, but underlying risk factors are poorly understood. This study aims to determine the incidence of children with suspected infection during mechanical ventilation and analyses risk factors for the finding of MDR Enterobacteriaceae in tracheal aspirates.

**Methods:**

A retrospective single-centre analysis of Enterobacteriaceae isolates from the lower respiratory tract of ventilated PICU patients from 2005 to 2014 was performed. Resistance status was determined and clinical records were reviewed for potential risk factors. A classification and regression tree (CRT) to predict risk factors for infection with MDR Enterobacteriaceae was employed. The model was validated by simple and multivariable logistic regression.

**Results:**

One hundred sixty-seven Enterobacteriaceae isolates in 123 children were identified. The most frequent isolates were *Enterobacter spp., Klebsiella spp.* and *E.coli.* Among these, 116 (69%) isolates were susceptible and 51 (31%) were MDR. In the CRT analysis, antibiotic exposure for ≥ 7 days and presence of gastrointestinal comorbidity were the most relevant predictors for an MDR isolate. Antibiotic exposure for ≥ 7 days was confirmed as a significant risk factor for infection with MDR Enterobacteriaceae by a multivariable logistic regression model.

**Conclusions:**

This study shows that critically-ill children with tracheal Enterobacteriaceae infection are at risk of carrying MDR isolates. Prior use of antibiotics for ≥ 7 days significantly increased the risk of finding MDR organisms in ventilated PICU patients with suspected infection. Our results imply that early identification of patients at risk, rapid microbiological diagnostics and tailored antibiotic therapy are essential to improve management of critically ill children infected with Enterobacteriaceae.

**Electronic supplementary material:**

The online version of this article (doi:10.1186/s12879-017-2251-x) contains supplementary material, which is available to authorized users.

## Background

Critically ill children are at high risk for severe healthcare associated infections (HAI) due to invasive devices and procedures, secondary immunosuppression and underlying diseases [1–3]. Multidrug-resistant (MDR) Gram-negative infections are an increasing threat to children admitted to the Paediatric Intensive Care Unit (PICU). However, predisposing factors for colonization and infection with MDR Gram-negative organisms are unclear in this vulnerable patient population.

MDR Enterobacteriaceae have become a particular concern for mechanically ventilated patients [4]. Placement of an endotracheal tube is followed rapidly by tracheal colonization with potentially pathogenic microorganisms from the oropharyngeal flora, including MDR organisms [5, 6]. Colonization of the lower respiratory tract by endogenous flora or opportunistic pathogens provides the major route to acquiring ventilator-associated pneumonia (VAP) [7]. Moreover, increased nasopharyngeal bacterial density is associated with a higher risk of invasive respiratory disease [8].

Data characterizing tracheal colonization in mechanically ventilated children independent of respiratory infection are scarce and vary substantially among hospitals and across countries as well as throughout the course of mechanical ventilation [9]. A clear predominance for Gram-negative organisms in ventilated PICU patients has been described in a paediatric colonization study in India [10]. Furthermore, endotracheal colonization was dominated by Enterobacteriaceae, in particular *E.coli* and *Enterobacter,* in two recent studies of VAP in adults and children [4, 11].

However, bacterial colonization does not necessarily imply infection and tracheal aspirates lack specificity for VAP [5]. Nevertheless, tracheal aspirates are part of the Center for Disease Control’s (CDC) criteria for the diagnosis of VAP and are frequently used to guide antibiotic therapy in PICU [12].

Empiric antibiotic treatment of Gram-negative infections is becoming increasingly difficult, because antibiotics that were previously considered the treatment of choice are no longer useful in MDR Gram-negative organisms [13–15]. If initial antimicrobial therapy is ineffective and only few treatment options remain in critically ill children, recurrence of infection, morbidity, mortality, length of PICU and hospital stay as well as healthcare costs will rise [13, 14]. Empiric antibiotic therapy might be improved by the identification of risk factors for colonization and infection with Enterobacteriaceae.

Risk factors for the acquisition of Enterobacteriaceae, especially extended-spectrum ß-lactamase (ESBL) producing organisms were investigated in adults during a stay in the intensive care unit. Major risk factors for infection due to ESBL-producing bacteria were: travel to high-prevalence countries, prior antibiotic use and mechanical ventilation [16]. Similar potential risk factors for infection due to MDR Enterobacteriaceae have been identified in neonates and children including the presence of chronic disease, previous hospitalization, invasive ventilation, pre-term low birth weight and antibiotic intake [17]. In particular, prior use of cephalosporins has been defined as an independent risk factor for the acquisition of MDR Enterobacteriaceae [18, 19]. Preceding antibiotic therapy may lead to a disruption of the intestinal flora and facilitate colonization and overgrowth of nosocomial MDR Gram-negative organisms [20]. These MDR strains increase the risk of infection by progressive colonization of the gastrointestinal and subsequently the respiratory tract during a hospital stay [21].

To our knowledge, clinical risk factors for infection of mechanically ventilated PICU patients with MDR Enterobacteriaceae have not been described to date. Knowledge of potential clinical risk factors for infection with MDR Enterobacteriaceae might help to initiate appropriate infection control precautions to prevent transmission of MDR bacteria in PICU and improve empiric antibiotic treatment.

Therefore, we aimed to determine the incidence and spectrum of MDR Enterobacteriaceae in children with suspected respiratory tract infection admitted to a large academic PICU from 2005 to 2014. Additionally, we analysed risk factors for the finding of MDR Enterobacteriaceae in these mechanically ventilated children.

## Methods

### Patient population and setting

A retrospective, single-centre analysis was performed in ventilated patients admitted to the PICU of the University Children’s hospital in Tübingen during the period from 2005 to 2014. This 12-bed PICU cares for critically ill infants and children with around 860 admissions per year. The main reason for admission is requiring cardiac surgery (46%), followed by general paediatric surgery (28%) and paediatric medical conditions that require intensive care treatment (22%). About 40 patients per year (5%) are transferred from long-term care facilities and have chronic conditions that require long-term tracheostomy. The organisational structure of this ward remained unchanged during the study period. Foreign-born patients from Eastern Europe and Senegal admitted for surgical and cardiothoracic procedures are an increasing part of the patient population in this PICU. Mechanically ventilated patients aged below 18 years with a tracheal aspirate positive for Enterobacteriaceae were identified from the institution’s Microbiology Database (HyBase Database, Cymed) and included in the study. Long-term ventilated children with tracheostomy were excluded. Medical records of these children were reviewed and demographic, clinical and microbiological data were extracted. Data obtained included age, sex, gestational age, birth weight, weight, height and BMI on admission. As exposures, we collected the following underlying conditions: pulmonary, cardiosurgical, neurological, gastroenterological, haematooncological disease and immunodeficiency, ventilated days before infection, days on ECMO and presence and days of a central venous line in situ before infection, duration of catecholamine therapy and days of antibiotic therapy in the 30 days before the positive culture result. Diagnosis of ventilator-associated pneumonia was made at the time of the positive tracheal culture result and defined according to the CDC criteria [12]. Secondary outcomes after infection were total ventilation days, PICU length of stay and all-cause mortality after 6 months. Microbiological data included the isolated organism in tracheal aspirate and a resistogram.

### Screening policy and infection control

On our PICU, children admitted from long-term care facilities, children with previous hospitalization within the last year or from high-prevalence countries are screened routinely for bacteria and resistances on admission. Screening includes a nasal swab for Methicillin-resistant *Staphylococcus aureus* (MRSA) since 2004 and a rectal swab for Vancomycin-resistant *Enterococci* (VRE) since 2012. During routine patient care, tracheal aspirates were taken from ventilated patients with clinical findings suggesting infection (including new onset of fever, rise in inflammatory markers or decline in oxygenation) or when tracheal aspirate had a purulent appearance. In case of isolation of an MDR organism, hygiene measures were set up according to the guidelines of the German Commission for Hospital Hygiene and Infectious Disease Prevention at the Robert Koch Institute and the local Hospital Hygiene Plan [22]. Isolation in a single room, contact and droplet precautions were performed to prevent transmission. To promote proper hand hygiene, an alcohol disinfectant is offered at each patient’s bedside. Patient to healthcare worker ratio was between 2:1 and 3:1.

### Mechanical ventilation

The mechanical ventilation system including humidification was set up by a Paediatric Intensive Care Nurse following standard procedures. Ventilatory parameters were set according to physician’s orders, adjustments were guided by routinely obtained blood gas analysis. Open or closed suctioning was performed as clinically indicated.

### Analysis of specimens

Specimens were collected through deep suctioning with the catheter passing beyond the endotracheal tube tip into the trachea or bronchi. The aspirate was routinely analysed in our hospital Microbiology Laboratory. Analysis was performed according to the local guidelines of the hospital Microbiology Laboratory. Organisms from tracheal aspirates were grown on agar plates or with liquid culture technique and incubated at 37 °C. Growth of organisms was monitored according to the local protocol. Susceptibility testing was realized by disc diffusion technique or the automated rapid susceptibility test system VITEK 2 (bioMérieux). To identify the organisms, the isolates underwent MALDI-TOF analysis or biochemical identification with the automated VITEK 2 system. Clinical breakpoints recommended by the European Committee On Antimicrobial Susceptibility Testing (EUCAST) were used to define susceptibility and resistance [23].

### Definition of infection and MDR

Infection was defined as the recovery of an Enterobacteriaceae isolate from the usually sterile respiratory tract. In this setting it was only possible to use the term “infection” to refer to both the situation in which infection was suspected with Enterobacteriaceae recovered from tracheal aspirate as well as to a respiratory disease (e.g., mild inflammation of the airways, tracheitis, ventilator-associated pneumonia etc.) that was attributed to the isolated microbe [24]. Resistance status was determined for each isolate according to the definition proposed by the European Society of Clinical Microbiology and Infectious Diseases for interim standard definitions for acquired resistance [25]. MDR was defined as acquired non-susceptibility to at least one agent in three or more antimicrobial categories with respect to intrinsic resistances.

### Statistical methods

To avoid bias due to multiple isolates in one patient, only the first isolate per patient was included. Patient data were analyzed using IBM SPSS Statistics Version 22 for Windows. Missing data points or data that were not applicable to the analysis were excluded. Statistical analysis was performed in consultation with the Department of Statistics/Biometrics of the University of Tübingen. Categorical variables between groups were compared with the [chi]^2^ test. Means of the two different groups were evaluated by two sample unpaired t-test if continuous variables were normally distributed. For intergroup comparison of continuous variables that were not normally distributed, the nonparametric Mann–Whitney U-Test was used. A *p*-value <0.05 was considered statistically significant. Results are presented as numbers for categorical variables. Normally and abnormally distributed quantitative variables are presented as mean ± standard deviation and median (minimum and maximum or interquartile range), respectively.

### Analysis of risk factors

Various studies have been performed to study risk factors for infection with MDR organisms in PICU patients [26–28]. Ten candidate risk factors, clinically relevant for infection with MDR organisms and available to study in our PICU setting, were selected from the literature [27–30]. These were patient age, duration of mechanical ventilation before infection, days of antibiotic pre-exposure, duration of presence of a central venous line, duration of catecholamine therapy, pre-existing gastroenterological, cardiac and pulmonary disease, length of PICU stay before culture and days on ECMO. In order to select suitable predictor variables for a final multivariable logistic regression model, we undertook a stepwise approach: First, a classification and regression tree (CRT) was employed to select the main risk factors for infection with MDR Enterobacteriaceae. The CRT approach is particularly applicable to find specific subgroups, relationships and cut-offs of continuous variables in larger sets of predictor variables that might not be detected with more common methods, often used in similar analyses (e.g. multivariable regression equations). These cut-off points can then be used to transform continuous into categorical variables for further analysis [31, 32]. Hence, we used CRT to select the input set of variables and to potentially find optimal cut-off points for categorisation of continuous variables in the following analysis. Second, we validated the findings with the common method of simple logistic regression, since the CRT model is a relatively uncommon statistical method. Odds ratio (OR) and 95% confidence interval (CI) were determined for each of the 12 risk factor variables. Third, variables found by the CRT model as well as variables with a *p*-value of <0.2 in the univariate analyses were selected as candidates for a multivariable logistic regression model. A stepwise backward selection process was used. In each step the variable with the least significant effect was eliminated. A cut-off of *α*_*crit*_ *<* 0.1 (“p-to-remove”) was set as a limit for removal of variables from the model. Statistics were all performed using SPSS (IBM SPSS Statistics Version 22 for Windows). A thorough explanation of the decision tree procedure, the model criteria and the command file for reproducing the classification tree are provided as Additional file 1 [33].

## Results

From 2011 to 2014, we observed a significant increase in the incidence of isolated Enterobacteriaceae specimens in tracheal aspirate compared to the beginning of the study period from 2005 to 2008 (mean 1.14 ± 0.55 vs 2.78 ± 0.56; *p* = 0.006). No MDR organisms were isolated in 2004, but 5 in 2014. However, there was high annual variability (see Additional file 2).

### Spectrum of isolates

During the whole study period, we obtained 167 Enterobacteriaceae isolates from the lower respiratory tracts of 123 intubated patients. 116 (69%) isolates were susceptible and 51 (31%) of all isolates were identified as MDR Enterobacteriaceae. The spectrum of isolates is shown in Additional file 3. *Enterobacter spp.* were the most prevalent genera (51 isolates, 30.5%), followed by *Escherichia coli* (47 isolates, 28.1%) and *Klebsiella spp.* (46 isolates, 27.5%). Most MDR organisms were *E.coli* (26/47 isolates), followed by *Klebsiella spp.* (13/46 isolates), *Enterobacter spp.* (7/51 isolates) and *Morganella* (4/6 isolates). The total number of ESBL producing organisms was 13 (7.8%) of all isolates. 7 out of 47 (14.9%) *E.coli* and 3 out of 46 (6.5%) *Klebsiella spp.* were ESBL-producing isolates.

### Patient characteristics and outcome

Patient characteristics and the clinical outcomes of 123 intubated children with Enterobacteriaceae infection are shown in Tables 1 and 2. Patients with MDR organisms did not differ from patients infected with susceptible organisms in sex, age, percentage of infants, gestational age, birth weight, BMI, presence and days with CVC in situ and days of antibiotic exposure during the last 4 weeks prior to the tracheal aspirate. Underlying diseases and conditions were similar between both groups with the exception of immunodeficiency, which was significantly more frequent in patients infected with an MDR organism. The clinical outcome was similar in children infected with an MDR versus a susceptible strain: children infected with Enterobacteriaceae had a median total ventilation time of 6 [IQR 3–19] and 8 [IQR 2–16] days, respectively. PICU length of stay was also similar in patients with MDR and susceptible strains (14 and 14.5 days). VAP occurred in 18 cases, resulting in a VAP incidence rate of 14%. With 9 VAP cases in both groups, this results in a VAP incidence rate of 11% among patients who were infected with a susceptible organism. Incidence rate of infection (21%) and all-cause mortality after 6 months (20%) were almost doubled in patients infected with MDR organisms. However this difference did not reach significance (*p* = 0.15, *p* = 0.22) (Tables 1 and 2).

**Table 1:** Characteristics of the source population and study population

	Source population (*n* = 7551)	Study population (*n* = 123)		
		MDR (*n* = 43)	Susceptible (*n* = 80)	*p*-value
Characteristics of the source and study population				
Sex m/f	4129/3422	24/19	50/30	0.47
Infant/Non-infant	974/6577	25/18	53/27	0.37
Age in years (median, [IQR])	3.3 [0.5;10.7]	0.4 [0.1;2.5]	0.6 [0.2;2.0]	0.94

Comparison between patients with MDR (*n* = 43) and susceptible (*n* = 80) isolates. *MDR* Multidrug-resistant Enterobacteriaceae, *IQR* Interquartile Range

**Table 2:** Anthropometric data and clinical outcome of the study population

	All (*n* = 123)	MDR (*n* = 43)	Susceptible (*n* = 80)	*p*-value
Gestational age in weeks (median, [IQR])	37 [34;39]	37 [34;39]	37 [33;39]	0.76
Birth weight (mean ± SD; kg)	2.62 ± 0.93	2.52 ± 1.05	2.67 ± 0.87	0.49
BMI (mean ± SD; kg/m^2^)	14.02 ± 4.21	13.65 ± 4.62	14.25 ± 3.95	0.47
Underlying diseases or conditions, n (%)				
Pulmonary	65 (53)	25 (58)	40 (50)	0.39
Cardiosurgical	80 (65)	27 (63)	53 (66)	0.70
Neurological	60 (50)	20 (47)	42 (53)	0.53
Gastroenterological	61 (50)	26 (61)	35 (44)	0.08
Hematooncological	10 (8)	3 (7)	7 (9)	0.73
Immunodeficiency	4 (3)	4 (9)	0 (0)	0.006
CVC in place, n (%)	62 (66)	24 (55)	38 (48)	0.48
CVC days (median, [IQR])	2.5 [0;8]	3 [0;9]	2 [0;7]	0.36
Days of antibiotic pre-exposure^a^ (median, [IQR])	2 [0;7]	4 [0;9]	2 [0;5]	0.2
Ventilated days (median, [IQR])	7 [3;18]	6 [3;19]	8 [2;16]	0.91
PICU length of stay (median, [IQR])	14 [7;32]	14 [6;32]	14.5 [7;33]	0.89
VAP Incidence, n (%)	18 (15)	9 (21)	9 (11)	0.15
All-cause mortality, 6 months, n (%)	17 (14)	8 (20)	9 (11)	0.22

Anthropometric data and clinical outcome of the study population of 123 intubated children with Enterobacteriaceae in tracheal aspirates. Comparison between patients with MDR (*n* = 43) and susceptible (*n* = 80) isolates. *MDR* Multidrug-resistant Enterobacteriaceae, *PICU* Paediatric Intensive Care Unit, *IQR* Interquartile Range, *BMI* body mass index, *CVC* central venous catheter, *VAP* ventilator-associated pneumonia

^a^Duration of antibiotic therapy up to 4 weeks prior to culture in days

### Patient characteristics and clinical outcome

**Fig. 1:**
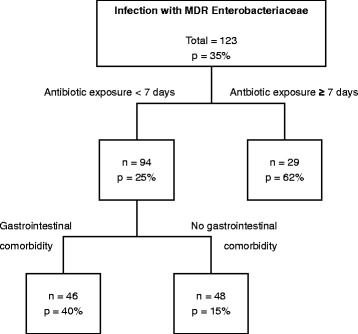
Classification and regression tree model to predict colonization with MDR Enterobacteriaceae. GI comorbidity = Gastrointestinal comorbidities including ileus, inborn abdominal anomalies, hepatopathies, necrotizing enterocolitis, gastritis and gastroenteritis

The CRT analysis (Fig. 1) revealed that length of antibiotic pre-exposure and presence of gastrointestinal comorbidity were the most relevant predictors for infection with an MDR strain. In this study, 62% of Enterobacteriaceae isolates were MDR if the duration of antibiotic pre-exposure was ≥7 days. Furthermore, the analysis revealed gastrointestinal comorbidity as a second predictive factor for MDR: In individuals infected with Enterobacteriaceae and a short duration of antibiotic exposure (<7 days), isolation of MDR strains was more likely if gastrointestinal comorbidity was present (40%). Conversely, isolation of susceptible strains was most likely in individuals without gastrointestinal comorbidity and with a short duration of antibiotic pre-exposure (<7 days) (85%). When evaluating the entire CRT model with both predictive parameters, the overall percentage of correct prediction was 71%, the risk of misclassification was 29%. Classification of susceptible strains was far more accurate (86% classified correctly) than classification of MDR strains alone (42% classified correctly). Normalised importance of the factors revealed days of antibiotic pre-exposure as the most relevant factor for MDR (100%), followed by gastrointestinal comorbidity (47%) (Additional file 4). These most relevant factors were included into a multivariable logistic regression analysis for further validation. According to the results of the CRT, antibiotic exposure was transformed into a categorical variable (antibiotic exposure for ≥7 days) for logistic regression analysis. Simple logistic regression analysis revealed antibiotic exposure for ≥7 days as a significant risk factor for infection with an MDR organism (OR 4.25; 95% CI 1.62-11.14). Gastrointestinal comorbidity did not reach significance as a potential risk factor for MDR in this analysis (OR 1.97; 95% CI 0.93–4.18) (Additional file 5). Variables found by the CRT model and variables with a *p*-value of <0.2 in the univariate analyses were selected as candidates for a multivariable logistic regression model. Candidates included antibiotic exposure for ≥7 days, gastrointestinal comorbidity and days on ECMO. After a backward elimination process, antibiotic exposure for ≥7 days and gastrointestinal comorbidity remained the most important risk factors for isolation of MDR Enterobacteriaceae. However, when entered into a multivariable logistic regression model (Table 3), gastrointestinal comorbidity did not reach significance as a predictive factor (OR 2.3; 95% CI 0.92–5.77). Antibiotic exposure for ≥7 days remained the only significant factor predicting MDR status in infected patients (OR 4.56; 95% CI 1.69–12.30).

**Table 3:** Multivariable logistic regression model predicting MDR status in infected patients. Estimates greater than 1 are associated with greater odds for tracheal infection with MDR Enterobacteriaceae

Risk factor	Adjusted Odds ratio	95% CI	*p*-value
Gastrointestinal comorbidity	2.3	0.92–5.77	0.08
Antibiotic pre-exposure ≥7 days	4.56	1.69–12.30	0.003

## Discussion

In this study we analysed the spectrum of Enterobacteriaceae in tracheal aspirates of intubated PICU patients from 2005 to 2014. The spectrum of Enterobacteriaceae in lower respiratory tract material revealed *Enterobacter spp., E.coli* and *Klebsiella spp.* as the most common isolates (86%). Comparable data of matching study settings are scarce in the current literature. Wilson et al. [5] collected daily tracheal aspirates from intubated children. Consistent with our findings, the most common Gram-negative organisms isolated were *Klebsiella spp.* and *E.coli*, followed by *Citrobacter freundii* and *Enterobacter cloacae*. Lee et al. [34] described the microbiological spectrum and susceptibility pattern of clinical isolates from a PICU and found a rate of 20% ESBL-positive *Klebsiella* in 2005. Our study displayed a lower rate with only 6.5% of *Klebsiella spp.* isolates being ESBL-positive. However, more than half of *E.coli* isolates (55%), about a quarter of all *Klebsiella spp.* (28%) isolates and 4 out of 6 *Morganella* (67%) isolates were MDR in the present study (Additional file 3). This finding is consistent with two large, nationwide studies of antibiotic drug use and bacterial resistance in the United States and in 53 German ICUs [35, 36]. In the latter, the most striking result was the ten-fold increase of 3^rd^ generation cephalosporin-resistant *E.coli* from 2001 to 2008. Highly resistant *E.coli* are known to have a 30% increase in infection rate and a higher mortality compared to susceptible isolates [37]. Keeping this in mind, MDR *E.coli* should alert PICUs to the prevalence of MDR *E.coli*.

In total, 167 Enterobacteriaceae isolates from lower respiratory tract samples were identified in 123 ventilated PICU patients. 43 (35%) patients were infected with MDR Enterobacteriaceae. Patient characteristics, including the proportion who were infants, days of antibiotic pre-exposure, presence of a CVC and underlying diseases did not differ between patients infected with susceptible versus MDR organisms. The only exception were patients with immunodeficiencies, who were significantly more likely to be infected with an MDR organism.

Clinical outcome of infection with MDR and susceptible Enterobacteriaceae was investigated in this study. Infection with MDR Gram-negative organisms has been associated with longer length of hospital stay or length of PICU stay [38]. We investigated whether patients infected with MDR organisms had more ventilated days in total, a longer length of PICU stay, a higher incidence of VAP or a higher all-cause mortality. Clinical outcome was similar in both groups in our study. This finding is to be expected, since MDR organisms commonly do not feature higher pathogenicity than their more susceptible counterparts of the same genera [39]. However, mortality from infection with MDR organisms is known to be higher due to the delay and a lower rate of appropriate empiric treatment [37, 40]. In our institution, susceptibility testing is rapidly available and empiric antibiotic therapy is rather aggressive. Differences in outcome may hardly be detectable in our study based on a low number of cases and the fact that PICU patients have several other underlying factors which may influence ventilated days, length of PICU stay, incidence of VAP and mortality.

Knowledge of potential clinical risk factors for infection with MDR organisms might help to improve infection control precautions, diagnostics and empiric antibiotic therapy. We investigated several factors described in the literature that could potentially increase the risk of infection with MDR Enterobacteriaceae [41, 42]. Of these potential risk factors, two were identified by the decision and regression tree analysis as the most important: (i) the duration of antibiotic exposure and (ii) gastrointestinal comorbidity were most relevant for infection with MDR Enterobacteriaceae.

Following the tree-based structure of the CRT analysis, antibiotic pre-treatment for ≥ 7 days increased the risk of infection with an MDR isolate to 60%. Furthermore, gastrointestinal comorbidity increased the chance of MDR infection from 25 to 40% in patients with a short (<7 days) duration of antibiotic therapy. It is well known that the stomach represents a reservoir for Gram-negative bacilli, especially in critically ill children who are fed by nasogastric tubes or treated with H (2) antagonists [43]. Transmission of pathogens to the lower respiratory tract may be facilitated in ventilated children with gastrointestinal comorbidities due to a higher rate of contamination of the hands or apparel of healthcare workers, contaminated respiratory equipment and micro-aspiration of stomach contents and flora into the lower respiratory tract. Long-term antibiotic use in these children (especially with cephalosporins) increases selective pressure on Gram-negative bacilli of the gastrointestinal and oropharyngeal flora, resulting in a higher rate of MDR Enterobacteriaceae [28].

Multivariable logistic regression supported the findings obtained in the CRT model with slight differences. Compared to the tree-based model, the adjusted odds ratio for gastrointestinal comorbidity did not reach significance. However, antibiotic exposure for ≥ 7 days remained the most relevant prognostic factors for infection with MDR Enterobacteriaceae. The OR of 4.56 (95% CI 1.69–12.30) for antibiotic exposure for ≥ 7 days indicates that after antibiotic therapy of 7 days or more, the risk of infection with MDR Enterobacteriaceae increases 4.56 times for every additional day of treatment. Our finding is consistent with numerous papers that have demonstrated antibiotic exposure as a strong risk factor for infection with MDR organisms [6, 18, 44]. Furthermore, this study investigated another important issue: namely, at which particular time an antibiotic might select MDR organisms in ventilated PICU patients. Our CRT analysis revealed a critical cut-off at 7 days of prior antibiotic treatment to increase the risk of infection with MDR Enterobacteriaceae. This cut-off at 7 days of antibiotic pre-treatment was validated by multiple logistic regression. In a previous study, children received antibiotics for clinician-suspected Ventilator-Associated Tracheitis (VAT) and the cut-off at 7 days of antibiotic therapy discriminated between short- and prolonged-course therapy, similar to our study. The hazard of colonization or infection with an MDR organism was more than 4 times greater if children had received a prolonged-course of antibiotic treatment, whereas short-course therapy had a significantly lower incidence of MDR infection and did not affect clinical outcome [45].

The present study has several limitations. First, this study was conducted in a retrospective observational manner and comprised a single PICU in a University Children’s Hospital. Therefore, data may be of limited applicability for PICUs in different settings. Second, assessment of risk factors for infection with MDR Enterobacteriaceae may have methodological deficits and may be biased with regards to estimates and associations. Selection bias towards infants and sicker patients may have affected our results. It is possible that tracheal aspirates were more likely to be taken in infants who were ventilated for a longer time after surgery for congenital heart or gastrointestinal defects. Consequently, infants might be overrepresented in our study. Additionally, sicker patients might have been more likely to be infected with an MDR pathogen. Furthermore, we could not exclude that the association of potential risk factors and infection with MDR Enterobacteriaceae was influenced by confounding factors. The patient population investigated in this study is a highly heterogeneous group with many potential confounders that are not uniform. Multiple hospitalisations, chronic diseases, congenital malformations, medication like H (2) blockers and antibiotic prophylaxis (e.g. with cephalosporins) may influence the rate of infection with MDR organisms. In particular, it is likely that severely ill children were treated with longer courses and a broader spectrum of antibiotics. These children may have a higher risk for infection with MDR organisms. Third, since there was no active screening policy for Enterobacteriaceae in tracheal aspirate on our ward, only patients with suspected infection were included into the study. Lastly, it should be noted, that the baseline classification odd (MDR versus susceptible organism) was 50%. Consequently, an overall predictive value of 71% of the CRT model implies a 21% increase in predictive accuracy.

## Conclusions

In summary, this study shows that *Enterobacter spp., Klebsiella spp.* and particularly *E.coli* are frequently isolated Enterobacteriaceae in lower respiratory tract materials in ventilated PICU patients. Gastrointestinal comorbidity may lead to a higher risk of infection with MDR isolates in these critically ill children. We demonstrate that prior use of antibiotics for ≥ 7 days significantly increases the risk of selection for MDR isolates in ventilated PICU patients infected with Enterobacteriaceae. Collectively, our results imply that early identification of patients at risk, rapid microbiological diagnostics and tailored antibiotic therapy are essential to improve management of critically ill children infected with Enterobacteriaceae.

## Additional files

Additional file 1: CRT explanation model criteria and command file. (DOCX 22 kb)
Additional file 2: Enterobacteriaceae isolates from 2005 to 2014. (DOCX 16 kb)
Additional file 3: Enterobacteriaceae distribution. Distribution of Enterobacteriaceae isolates (*n* = 167) in lower respiratory tract material, MDR (*n* = 51) vs susceptible (*n* = 116) organisms during the study period. (XLSX 14 kb)
Additional file 4: Risk estimate and classification model. (DOCX 14 kb)
Additional file 5: Simple logistic regression. (DOCX 12 kb)

## Abbreviations

BMI: Body mass index
CDC: Centers for Disease Control and Prevention
CRT: Classification and regression tree
ECMO: Extracorporeal membrane oxygenation
ESBL: Extended spectrum ß-lactamases
EUCAST: European Committee On Antimicrobial Susceptibility Testing
HAI: Hospital acquired infection
ICU: Intensive Care Unit
IQR: Interquartile range
MDR: Multidrug-resistant
OR: Odds ratio
PICU: Paediatric Intensive Care Unit
VAP: Ventilator-associated pneumonia
VAT: Ventilator-Associated Tracheitis
