# Supplementary material for: Suspicion of respiratory tract infection with multidrug-resistant Enterobacteriaceae: epidemiology and risk factors from a Paediatric Intensive Care Unit
Source: BMC Infect Dis. 2017 Feb 21;17:163. doi: 10.1186/s12879-017-2251-x (PMC5320655; doi:10.1186/s12879-017-2251-x)
Supplement: Additional file 1: — CRT explanation model criteria and command file. (DOCX 22 kb) [file 12879_2017_2251_MOESM1_ESM.docx]

Additional file 1: CRT explanation, model criteria and command file

**Classification and regression tree analysis (CRT)**

The decision tree procedure creates a tree-based classification model to predict values of a target variable (dependent) based on independent predictor variables [[31](#_ENREF_31)]. Based on statistical significance, the algorithm determines a set of predictor variables via binary partition. The result is a classification model consisting of a root node representing the dependent variable and several branches and nodes. Each node is assigned to one class representing the most appropriate target variable. Finally, the *if-*then spilt and stop conditions of the most relevant candidate predictors result in terminal nodes that provide an accurate prediction of the dependent target variable. In consideration of the sample size, adequate default values of 30 cases for parent nodes and 15 cases for child nodes were chosen. We applied 25-fold cross-validation to avoid over-fitting and to increase the predictive accuracy of the model; maximum tree depth was 5, significance was set at 0.01.

**Model criteria:**

**potential risk factors for colonization with MDR Enterobacteriaceae**

| **Potential risk factor** | **Name used in the command file** |
| --- | --- |
| Age | AlterimUntersuchungsjahr [s] |
| Treatment with catecholamines 4 weeks before or after obtaining the isolate | KatecholamineTage4wochenpräundpostCx [s] |
| Mechanical ventilation before obtaining the isolate (in days) | BeatmunginvasivvorCxTagenuraufPICU [s] |
| Days of presence of a central venous line before obtaining the isolate | ZVKpräCx_dnuraufPICU [s] |
| Gastroenterological comorbidity | GastroZusatzerkr [n] |
| Cardiac comorbidity | KardialeErk [n] |
| Pulmonary comorbidity | PulmonaleErk [n] |
| Length of stay on the PICU | PICULOSpräCx [s] |
| Days on Extracorporeal life support | ECMOTage [s] |
| Days of Antibiotic treatment before obtaining the isolate | DauerdAntibiot.Behandlung  vorCxTagemax4WochenimVoraus [s] |

**Command file (IBM SPSS Statistics Version 22 for Windows):**

TREE MDRstatus [n] BY AlterimUntersuchungsjahr [s]

KatecholamineTage4wochenpräundpostCx [s] BeatmunginvasivvorCxTagenuraufPICU [s]

ZVKpräCx_dnuraufPICU [s] GastroZusatzerkr [n] KardialeErk [n] PulmonaleErk [n] PICULOSpräCx [s]

DauerdAntibiot.BehandlungvorCxTagemax4WochenimVoraus [s] ECMOTage [s]

/TREE DISPLAY=TOPDOWN NODES=STATISTICS BRANCHSTATISTICS=YES NODEDEFS=YES SCALE=AUTO

/DEPCATEGORIES USEVALUES=[.0 1.0] TARGET=[1.0]

/PRINT MODELSUMMARY IMPORTANCE CLASSIFICATION RISK

/GAIN CATEGORYTABLE=YES TYPE=[NODE] SORT=DESCENDING CUMULATIVE=NO

/SAVE PREDVAL PREDPROB

/METHOD TYPE=CRT MAXSURROGATES=AUTO PRUNE=NONE

/GROWTHLIMIT MAXDEPTH=AUTO MINPARENTSIZE=30 MINCHILDSIZE=15

/VALIDATION TYPE=CROSSVALIDATION(25) OUTPUT=BOTHSAMPLES

/CRT IMPURITY=TWOING MINIMPROVEMENT=0.001

/COSTS EQUAL

/PRIORS FROMDATA ADJUST=NO

/MISSING NOMINALMISSING=MISSING.
