# Supplementary material for: Suspicion of respiratory tract infection with multidrug-resistant Enterobacteriaceae: epidemiology and risk factors from a Paediatric Intensive Care Unit
Source: BMC Infect Dis. 2017 Feb 21;17:163. doi: 10.1186/s12879-017-2251-x (PMC5320655; doi:10.1186/s12879-017-2251-x)
Supplement: Additional file 2: — Enterobacteriaceae isolates from 2005 to 2014. (DOCX 16 kb) [file 12879_2017_2251_MOESM2_ESM.docx]

Additional file 2: Enterobacteriaceae isolates from 2005-2014

**Enterobacteriaceae in lower respiratory tract material –**

**Incidence rate of susceptible and MDR Enterobacteriaceae**

**isolates from 2005-2014**

Incidence rate of infection per year with susceptible and MDR Enterobacteriaceae in patients admitted to PICU from 2005-2014. A comparison of the two 4-year episodes 2005-2008 and 2011-2014 shows a significant increase in total Enterobacteriaceae incidence rate (mean 1.14±0.55 vs 2.78±0.56; p=0.006), susceptible Enterobacteriaceae incidence rate (mean 0.9±0.52 vs 1.79±0.24; p=0.021) and MDR Enterobacteriaceae incidence rate (mean 0.24±0.19 vs 0.93±0.43; p=0.025).
