# Supplementary material for: Suspicion of respiratory tract infection with multidrug-resistant Enterobacteriaceae: epidemiology and risk factors from a Paediatric Intensive Care Unit
Source: BMC Infect Dis. 2017 Feb 21;17:163. doi: 10.1186/s12879-017-2251-x (PMC5320655; doi:10.1186/s12879-017-2251-x)
Supplement: Additional file 4: — Risk estimate and classification model. (DOCX 14 kb) [file 12879_2017_2251_MOESM4_ESM.docx]

Additional file 4: Risk estimate and classification model

**Risk estimate and classification model**

Risk estimate and classification of MDR and susceptible Enterobacteriaceae in tracheal aspirate. The table shows the number of cases classified correctly and incorrectly for each category of the dependent variable. For susceptible organisms, the model predicts 69 cases (86%) correctly being susceptible. 11 of the susceptible organisms are inaccurately classified as MDR. Likewise, for MDR organisms, the model predicts 18 cases (42%) correctly being MDR. 25 of the MDR organisms are inaccurately classified as susceptible. The model classifies 71% of cases correctly and is more accurate to predict susceptible (86% correct) than MDR (42% correct) organisms.

| **Classification** |  |  |  |
| --- | --- | --- | --- |
| ***Observed*** | ***Predicted*** | | |
|  | ***Susceptible*** | ***MDR*** | ***Percent correct*** |
| Susceptible | 69 | 11 | 86.3% |
| MDR | 25 | 18 | 41.9% |
| Overall percentage | 76.4% | 23.6% | 70.7% |
| Growing method: CRT  Dependent variable: MDR | | | |

**Model importance by independent predictor variables**

Ranks for each independent predictor variable according to its importance (%) to the model are described in the following table.

| **Independent predictor variables** | **Normalised predictor importance** |
| --- | --- |
| Days of antibiotic pre-exposure (d) | 100% |
| Gastroenterological comorbidity | 47% |
| Days on ECMO (d) | 23% |
| Ventilated days (d) | 19% |
| Days of catecholamine therapy | 19% |
| Days of CVC (d) | 19% |
| PICU length of stay (d) | 17% |
| Age | 2% |
| Pulmonary comorbidity | 2% |
| Cardiac comorbidity | 1% |
