# Supplementary material for: Suspicion of respiratory tract infection with multidrug-resistant Enterobacteriaceae: epidemiology and risk factors from a Paediatric Intensive Care Unit
Source: BMC Infect Dis. 2017 Feb 21;17:163. doi: 10.1186/s12879-017-2251-x (PMC5320655; doi:10.1186/s12879-017-2251-x)
Supplement: Additional file 5: — Simple logistic regression. (DOCX 12 kb) [file 12879_2017_2251_MOESM5_ESM.docx]

Additional file 5: Simple logistic regression

**Simple logistic regression predicting MDR status in infected patients.**

Estimates greater than 1 are associated with greater odds for tracheal infection with MDR Enterobacteriaceae.

| **Risk factor** | **Odds ratio** | **95% CI** | **p-value** |
| --- | --- | --- | --- |
| Antibiotic pre-exposure ≥7 days | 4.25 | 1.62-11.14 | 0.003 |
| Gastrointestinal comorbidity | 1.97 | 0.93-4.18 | 0.08 |
| Ventilated days (d) | 1.02 | 0.97-1.1 | 0.44 |
| Age (years) | 1.0 | 0.93-1.09 | 0.93 |
| PICU length of stay (d) | 1.0 | 0.99-1.03 | 0.54 |
| CVC days (d) | 1.01 | 0.99-1.04 | 0.26 |
| Days of catecholamine therapy (d) | 0.99 | 0.94-1.04 | 0.63 |
| Cardiac comorbidity | 0.86 | 0.4-1.86 | 0.7 |
| Pulmonary comorbidity | 1.39 | 0.66-2.93 | 0.39 |
| Days on ECMO (d) | 1.31 | 0.92-1.85 | 0.13 |
